# Understanding the bacterial compositional network associations between oral and gut microbiome within healthy Koreans

**DOI:** 10.1080/20002297.2023.2186591

**Published:** 2023-03-03

**Authors:** Jinuk Jeong, Kung Ahn, Seyoung Mun, Kyeongeui Yun, Yeon-Tae Kim, Won Jung, Kyung Eun Lee, Moon-Young Kim, Yongju Ahn, Kyudong Han

**Affiliations:** a Department of Bioconvergence Engineering, Dankook University, Yongin, Republic of Korea; b Department of Human microbiome research HuNbiome Co. Ltd, R&D Center, Seoul, Republic of Korea; c Department of Microbiology, College of Science & Technology, Dankook University, Cheonan, Republic of Korea; d Center for Bio‑Medical Engineering Core Facility, Dankook University, Cheonan, Republic of Korea; e Department of Periodontology, Daejeon Dental Hospital, Institute of Wonkwang Dental Research, Wonkwang University College of Dentistry, Daejeon, Republic of Korea; f Department of Oral Medicine, School of Dentistry, Jeonbuk National University, Jeonju, Republic of Korea; g Department of Oral and Maxillofacial Surgery, College of Dentistry, Dankook University, Cheonan, Republic of Korea

**Keywords:** Next-generation sequencing, metagenome sequencing, Korean oral microbiome, oral–gut association microbial type, bacterial co-occurrence networks

## Abstract

Oral microbial ecosystem could influence intestinal diseases, but there have been insufficient studies demonstrating the association of microbial composition between the oral cavity and the intestinal system. Thus, we aimed to investigate the compositional network within the oral microbiome related to gut enterotype from saliva and stool samples collected from 112 healthy Korean subjects. Here, we performed bacterial 16S amplicon sequencing from clinical samples. Then, we determined oral microbiome type related to individual’s gut enterotype for healthy Korean. The co-occurrence analysis was performed to interactivity prediction of microbiome within saliva samples. As a result, it could be classified into two Korean oral microbiome types (KO) and four oral–gut-associated microbiome types (KOGA) according to distribution and significant differences of oral microflora. The co-occurrence analysis showed various bacterial compositional networks linked around *Streptococcus* and *Haemophilus* within healthy subjects. The present study was first approach in healthy Koreans to identify the oral microbiome types related to the gut microbiome and investigate their characteristics. Hence, we suggest that our results could be potential healthy control data for identifying differences in microbial composition between healthy people and oral disease patients and studying microbial association with the gut microbial environment (oral–gut microbiome axis).

## Introduction

Next-generation sequencing technology (NGS)-based metagenome profiling classifies the microbial community present in a human living environment, and it is a research method that can predict various biological phenomena generated there in connection with the colonization of different microbial species [1,2]. It has also been used to reveal the association between microbes and human health by defining microbial communities that exist inside and outside the human body, such as the intestinal mucosa, oral, and skin [3,4]. The National Institutes of Health (NIH) Human Microbiome Project (HMP) consortium explained that dysbiosis, which is a microbial imbalance, is a major cause of several human diseases, including cancer, obesity, inflammatory bowel disease, and periodontal disease [5–7]. The HMP recruited large healthy adult populations to derive these findings and established an NGS-based microbiome database for each body site of interest, such as the vagina, gut, and oral tract, using clinical specimens obtained from these populations. Through the project, the HMP has publicly provided the microbial taxonomic databases of healthy adults to microbial researchers, and efforts to modify and update the taxonomic classification database on human microbiome communities have continued with the development of various metagenomic sequencing platforms [8,9]. Recently, many researchers have become interested in using the human microbiome database explained by these large-scale microbiome consortiums to identify the connection between microbiota in multiple body sites rather than in specific body sites. Many researchers have continued to investigate the effect of a network between gut and oral microbiome compositions on host health.

The oral cavity is the starting point of the digestive and respiratory organs, and saliva especially contains many suspended microbial substances that can reach the intestinal tract and affect microbial diversity [10]. Unlike the intestinal tract where most anaerobic bacterial species inhabit, aerobic bacteria dominate within the oral cavity through colonization, such as biofilm formation on the teeth, gums (supra- and sub-gingival sites), and oral mucosal surfaces [11]. Metabolites produced from most anaerobic bacteria in the human body are highly involved in maintaining the metabolic balance and homeostasis of the host. However, changes in the ratio of some anaerobic species in the oral cavity can disrupt these balances [12,13]. Previous studies showed that the intraoral frequency of *Porphyromonas gingivalis*, one of the ‘red complex’ bacteria associated with periodontal disease, is involved in changes in the intestinal *Bacteroidetes*/*Firmicutes* ratio [14,15]. Several studies reported that changes in the host’s intestinal *Bacteroidetes*/*Firmicutes* ratio could lead to obesity and alter the balance of inflammatory responses and insulin resistance [16,17]. In addition, microbiome studies on the effects of changes in the composition and diversity of various oral microorganisms, such as *Selenomonas noxia*, on the health status of many human body sites, such as the lungs, appendix, and intravenous sites, are ongoing [14,18]. As such, the importance of research has been highlighted because the various microbial communities living in the oral cavity affect the balance of intestinal microorganisms and are more broadly and closely related to the biological metabolisms of the host body.

In this study, the oral microbiome types of healthy Koreans without oral diseases were classified by their associations with the gut microbiome types of individuals through comprehensive 16S microbiome analysis at the genus level. In addition, we aim to describe the intra-oral clinical environment for each oral microbiome type as its association with changing patterns of relative bacterial frequency by identifying the compositional correlative relationship among bacterial genera within each classified type. Ultimately, through this novel approach, which determines oral microbiome types that reflect individual gut microbiome compositions, we aimed to provide healthy control data for identifying differences in the microbial composition between healthy people and patients with oral diseases, such as periodontitis or dental caries, and studying microbes associated with the gut microbial environment (oral–gut microbiome axis).

## Materials and methods

### Clinical information of participants in this study

All clinical experiments (recruitment of participants and clinical samples collection) conducted for this study were approved by the Institutional Review Board of Dankook University Hospital (IRB numbers: 2020-10-015). Of those 22 years or older who visited the Department of Periodontics, 112 patients without oral disease participated in this study (Table 1). Each participant was classified into healthy oral group after being tested for oral environmental conditions and whether or not it is a dental disease through a simple survey and interview including medical history with the dentist prior to a clinical study. The criteria for exclusion from the clinical test were as follows: patients who refused to participate in this study, with severe mental disorders, had systemic diseases with potential dental disease effects, drug abuse, were pregnant, had taken took antibiotics within the past 6 months, or had active dental treatments (including scaling, root planning). All clinical examinations on healthy subjects were performed by a dentist, in which pocket depth (PD), clinical attachment loss (CAL), gingival index (GI), and plaque index (PI) were measured through the full arch.

**Table 1.** Clinical information of experimental participant group.

Characteristic	Participants
**Sex**	
Male	67
Female	45
**Age (years)**	38.11^a^ ± 15.15
**Clinical samples**	
Gut (stool sample number)	112
Oral (saliva sample number)	112
**Clinical examination**	
PD (mm)	2.08 ± 0.51
CAL (mm)	2.14 ± 0.59
GI	0.15 ± 0.47
PI	0.42 ± 0.71

^a^Values are presented as mean ± standard deviation.

PD, probing depth; CAL, clinical attachment loss; GI, gingival index; PI, plaque index.

### Clinical sample collection and metagenomic DNA extraction

The two types of clinical specimens (saliva and stool) were collected in each pair per individual to confirm the oral and gut microbial composition through a non-invasive sampling method. All specimens were collected using the NBgen-GUT NP self-collection tube, which included microbial preservation solution (Noble Biosciences, Republic of Korea). All participants were requested to avoid eating food and oral hygiene (such as brushing or flossing teeth) at least 2 h before sampling. All collected clinical specimens were used to extract metagenomic DNA (mDNA) for 16S microbiome analysis. The mDNA was extracted using the QIAamp PowerFecal Pro DNA Kit (QIAGEN, Germany), and all experimental processes were performed according to the formal protocol guide provided in the Kit. The quality and yield of the extracted mDNAs were confirmed using a Bioanalyzer (Agilent 2100, USA) equipment at the Center for Bio-medical Engineering Core Facility (Dankook University, South Korea) and stored at 4°C until the following process.

### Illumina library construction and 16S V3-V4 sequencing

A total of 224 16S V3-V4 amplicon libraries (112 samples per each sampling site) were prepared following the Illumina metagenomic sequencing library construction workflow. The metagenome sequencing platform of the Illumina targeted a partial area containing the V3-V4 hypervariable region of the bacterial 16S rRNA gene. PCR for amplifying the target region was performed using the KAPA HiFi Hot Start Ready Mix (2×) (Roche, Mannheim, Germany), and a pair of 16S V3-V4 target-specific primer recommended by Illumina. The primer sequences were as follows: 16S 341F forward primer is 5’-TCGTCGGCAGCGTCAGATGTGTATAAGAGACAGCCTACGGGNGGCWGCAG-3“ and 16S 805 R reverse primer is 5”-GTCTCGTGGGCTCGGAGATGTGTATAAGAGACAGGACTACHVGGGTATCTAATCC-3’. After the PCR amplification, the purification process of all PCR amplicon products was conducted using the AMPure XP beads (Beckman Coulter, USA). Then, additional PCR amplification was performed using Nextera XT Index Kit (Illumina, USA), which included Illumina multiplexing dual index barcode and sequencing adapter sequence. The final PCR products were then purified once again using the AMPure XP beads. After the amplicon library construction, the 16S metagenome sequencing was performed using the paired-end 2 × 300 bp Illumina MiSeq^TM^ protocol (Illumina Miseq, USA).

### 16S microbiome data analysis

16S V3-V4 sequencing raw read data were demultiplexed by using the split_libraries_fastq.py function in the QIIME2 microbiome data analysis pipeline, and quality sequences were trimmed using the Divisive Amplicon Denoising Algorithm 2 (DADA2), which detects and corrects amplicon errors and filters out PhiX and chimeric sequences, in R (version 3.3.2). The R analysis parameters were as follows: EE = 2, TruncL= c (200, 180), and q = 10. At the same time, the high-quality sequencing data that completed the quality check was used to cluster the Amplicon Sequence Variants (ASVs) using the QIIME2-based DADA2 pipeline. The beta diversity analysis to identify the difference in microbial composition between each comparison group was estimated using Principal Coordinate Analysis (PCoA) based on the unweighted_UniFrac (considering phylogeny), Bray-Curtis (considering microbial abundance), and Jensen–Shannon divergence (in PAM clustering method) distance dissimilarity estimation matrices. Finally, the representative ASV data, generated after denoising, was used to identify bacterial taxonomy using a sklearn-based Naive Bayes classifier trained on the SILVA v138 99% bacterial 16S V3-V4 database. In this process, the feature table assigned to the bacterial taxonomy for each ASV data was formed by setting a confidence threshold of 70% (default) or more value (Supplementary Table S2). Then, the bacterial relative abundance analysis at each biological taxonomic rank was performed to determine the relative frequency of bacterial strains present in each sample to identify differences in the bacterial composition of each group (Supplementary Table S3).

### Microbiome type clustering

To create clusters of the Korean oral microbiome type (KO type) and the Korean oral–gut-associated oral microbiome type (KOGA type), the bacterial genera, which were classified by more than 0.01% relative frequency within healthy subjects (Supplementary Table S3), were preferentially filtered from the relative abundance data classified from each sample. This process is essential to distinguish bacterial genera classified high-relative frequency, which plays a dominant role in discriminating microbial clusters within each sample of healthy subjects group. Based on the relative frequency of the filtered bacterial taxa (at the genus level), the dissimilarity score of bacterial composition for each sample was calculated using the ‘Jensen–Shannon divergence (JSD)’ distance clustering metric. Subsequently, estimating the optimal number of bacterial composition clustering for each sample and evaluating the robustness of the clusters was performed by applying the ‘Calinski–Harabasz (CH)’ index and Silhouette validation method included in the R studio’s ClusterSim library. Finally, the ‘Partitioning Around Medoid (PAM)’ clustering method was applied to cluster each sample according to the optimal number of clusters calculated previously. The KOGA type was designated by applying the same clustering pipeline by correlating the gut microbiome data of samples corresponding to each KO type previously clustered. The Microbiome Multivariable Association with Linear Models (MaAsLin 2) were used to cross-validate about PAM clustering results (Supplementary Fig. S2). The data analysis process using the MaAsLin2 was as follows: (1) The unnamed genera (or unclear nomenclature of bacterial taxa) with ‘;__’ were removed (Database: SILVA 138 v, Naïve Bayes classification). Then, the bacterial genus abundance profiles were normalized to generate probability distribution. (2) The bacterial genera with very low relative abundance were removed to decrease the noise, if their average abundance across all samples was below 0.01%. (3) Finally, the preprocessed input data was applied to BiocManager’s MaAsLin 2 package in R studio. In the MaAsLin 2 analysis, relative abundance data created by QIIME2 was used as input data. So, normalization and prevalence filtering about input data were set to ‘None’ parameter in MaAsLin 2 package in R studio.

### Compare with public oral microbiome data in other nations

The oral microbiome data in other nations (China-Taiwan, Portugal-Spain, and Australia) annotated on the NCBI database was used to compare the differences with the oral microbiome type classified in the present study. The sources (Bioproject number on NCBI database) of public data applied in this comparison were as follows: China-Taiwan_PRJNA503603 (76 samples), PRJNA609009 (21 samples), and PRJEB39064 (27 samples); Portugal-Spain_PRJNA774299 (22 samples) and PRJNA427101 (1319 samples); Australia_PRJNA558132 (64 samples).

### Co-occurrence and correlation analysis

Co-occurrence analysis was performed to identify other bacterial genera forming a compositional network with each dominant bacterial genera of KO type. In this process, the ‘Sparse Correlation Network Investigation for Compositional Data (SCNIC)’ analysis-based ‘SparCC’ correlation method [19] was applied using the bacterial ASVs frequency data (not considering relative abundance), and the parameters set for data analysis were as follows: cutoff threshold of the SparCC correlation value (*R* = SparCC correlation coefficient): |R| > 0.3 and *p*-value <0.05. The output data generated through the co-occurrence analysis was visualized using the ‘Cytoscape’ bioinformatics tool and the ‘networkx’ tool using the python operating system.

### LEfSe analysis

The Linear discriminant analysis Effect Size (LEfSe) analysis was performed to identify distinct oral bacterial genera or species showing a significant difference in relative bacterial frequency within each KOGA type sub-classified from KO type. The threshold on the logarithmic LDA score for discriminative features was set to 2.0 (indicating significant differential abundance).

### Scoring for characterization of KOGA type

First, we converted the relative frequency of each selected bacterial species into a *z*-score to confirm its standard normal distribution within each KOGA type. Next, we converted each calculated *z*-score value into a percentile value (%) and estimated the potential health characteristic of each KOGA type by performing a scoring process based on the quartile formula. In this analysis, since the characteristics of each type based on scoring were standardized to the distribution rate of beneficial bacteria, the scoring process for harmful bacteria was performed by inversely converting the negative and positive signs of each calculated *z*-score value.

### Statistical analysis

The Mann–Whitney *U* statistical test was applied to comparative statistical analysis between all comparison groups established for this study. The permutational multivariate analysis of variance (PERMANOVA) non-parametric statistical test was applied to identify the statistical significance of beta-diversity analysis using the Bray–Curtis, unweighted_UniFrac, and Jensen–Shannon divergence (JSD) distance matrices. The Kruskal–Wallis ANOVA test and Mann–Whiteny *U* test were applied to LEfSe analysis. The Statistical significance evaluated from all comparison set was denoted as asterisk (*) if *p*-value<0.05, (**) if *p*-value<0.01, and (***) if *p*-value<0.001.

## Results

### 16S metagenomic sequencing data processing of two different clinical sample types

We comprehensively investigated the bacterial compositional network associations between oral and gut microbiomes by clustering healthy KO types and KOGA types. We checked the integrity of the metagenomic DNA (mDNA) samples extracted from the saliva (oral) and stool (gut) samples of 112 participants in this study, because high-quality mDNA extraction can affect accurate microbial quantification. For the comprehensive 16S microbiome analysis, we performed 16S V3-V4 metagenomic sequencing using constructed sequencing libraries. As a result of the sequencing, the average demultiplexed read counts generated from the oral and gut samples were 47,675 and 59,042, respectively (Suplementary Table S1). Finally, the average high-quality raw read counts (non-chimeric reads) used for the comprehensive 16S microbiome analysis after the filtering process were 32,004 and 35,233, respectively. For comprehensive microbiome analysis, the ASV taxonomy classification numbers of the oral and gut sample types clustered with >70% (default) confidence threshold about the sequence alignment with the SILVA v138 16S rRNA reference database were 4,123 and 4,354, respectively (Supplementary Table S2). Our raw 16S V3-V4 sequencing data processing and ASV taxonomic clustering results allowed for subsequent microbiome analyses based on high-quality sequence data.

### Clustering of healthy Korean oral microbiome type

**Figure 1.**
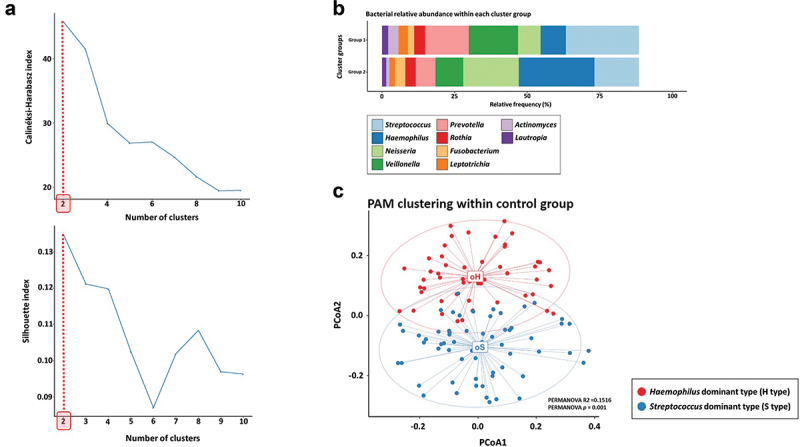
Data processing for clustering KO type using PAM clustering method.

**Note**: Data processing showing the determination of Korean oral microbiome type (KO type) clustered within the oral (saliva) samples of 112 healthy Korean based on the PAM clustering method. (a) Line graphs showing the results of the Partitioning Around Medoid (PAM) clustering method using the Calinéksi–Harabasz and Silhouette indexes based on the distance dissimilarity score of bacterial community between each individual within the healthy subjects calculated by applying the Jensen–Shannon divergence (JSD) formula. The horizontal axis on the graphs represents the optimal number of microbial clustering identified within the group. (b) Relative abundance bar plot showing the bacterial composition of each divided microbial cluster group within the healthy subjects by using the PAM clustering method. Bar graph and legend box on the plot show relative proportion and each taxon name of the top 10 bacterial genera in each cluster group. (c) This Principal Coordinate Analysis (PCoA) plot shows that the major dominant bacterial genera identified within each sample corresponding to the two different cluster groups are *Streptococcus* and *Haemophilus* (*oH and oS on the plot: S type in oral samples and H type in oral samples).

To cluster the dominant bacterial types in the oral microbiome in the saliva samples of healthy Koreans (KO type), we applied relative bacterial abundance data at the genus level for healthy subjects (*n* = 112; Supplementary Table S3). Prior to this analysis, we filtered 75 bacterial taxa calculated to an average relative frequency of over 0.01% from the 215 bacterial genera classified from the healthy subjects to decrease the analytical noise. Based on the distance dissimilarity score of the bacterial communities between each individual calculated by applying the Jensen–Shannon divergence (JSD) formula, we confirmed that the optimal cluster number was two (Figure 1a). Next, based on the data in Supplementary Table S3, we identified that the major dominant bacterial genera corresponding to each optimal cluster group estimated by PAM clustering were *Streptococcus* and *Haemophilus* (Figure 1b). When performing principal coordinate analysis (PCoA) based on JSD distance score values, the clusters could be divided into two groups, the *Streptococcus*-dominant group: *n* = 62/112_55.4% and the *Haemophilus*-dominant group: *n* = 50/112_44.6% (*p* < 0.01; Figure 1c). *Streptococcus* and *Haemophilus* are genera that include many bacterial species corresponding to normal microflora in the oral cavity of healthy humans and are known to form a biological network with other oral microbiota and the host [10,20,21].

### Determining healthy Korean oral–gut-associated oral microbiome type

**Figure 2.**
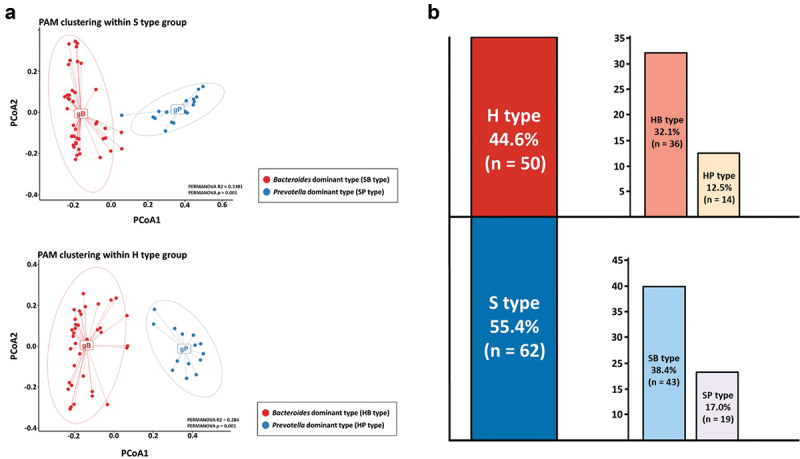
Data process of KOGA type clustering and comparison with national oral microbiome data.

**Note**: These figures show the process of clustering Korean oral–gut-associated oral microbiome type (KOGA type) that reflects the gut enterotype of each individual corresponding to previously clustered KO types and show the results of comparing the KO type with the oral microbiome data of other living areas. (a) PCoA plot showing that gut enterotype of each sample within two different KO types was divided into *Bacteroides* and *Prevotella* dominant types through the PAM clustering method (*gB and gP on the plot: B and P types in gut samples of each KO type). (b) Bar graph showing the proportion of subjects in the control group corresponding to the KO and KOGA types clustered in the present study.

To determine the oral microbiome types that could explain the compositional association with intestinal microbiome (KOGA type; Korean oral–gut-associated oral microbiome type) within each KO type (*Streptococcus*-dominant oral microbiome type: S type; *Haemophilus*-dominant oral microbiome type: H type) previously clustered, we applied the relative abundance analysis data for the gut microbiome of individuals corresponding to each KO type (Supplementary Table S4). This approach was performed by applying the same workflow as the clustering method for the KO type. We confirmed that the optimal cluster number between each gut microbial composition within the group corresponding to the S and H types was two, respectively (Supplementary Fig. S1A). Then, based on the data in Supplementary Table S4, we found that the major dominant bacterial genera that formed the cluster groups within each KO type group were *Bacteroides* and *Prevotella* (Supplementary Fig. S1B). Figure 2a shows that clustering was formed between inter-individuals with microbial compositions classified the relative bacterial frequency of *Bacteroides* and *Prevotella* as dominant bacterial genera within the group of each KO type. The results of several previous studies that conducted comprehensive 16S microbiome analyses showed that the healthy Korean enterotype was predominantly clustered as *Bacteroides* and *Prevotella* types [22–24]. Based on this result, we suggest that our findings are not significantly different from the existing reference database associated with Korean microbiome research. Finally, we identified four KOGA types from 112 healthy subjects; SB (oral_*Streptococcus*-gut_*Bacteroides* dominant microbiome type (*n* = 43), SP (oral_*Streptococcus*-gut_*Prevotella* dominant oral microbiome type; *n* = 19), HB (oral_*Haemophilus*-gut_*Bacteroides* dominant oral microbiome type; *n* = 36), and HP (oral_*Haemophilus*-gut_*Prevotella* dominant oral microbiome type; *n* = 14) types were divided into 38.4, 17.0, 32.1, and 12.5%, respectively (Figure 2b). These enterotype clustering results within the oral and gut microbiome data showed the same pattern in the Microbiome Multivariable Association with Linear Models analysis (MaAsLin 2; Supplementary Fig. S2).

**Figure 3.**
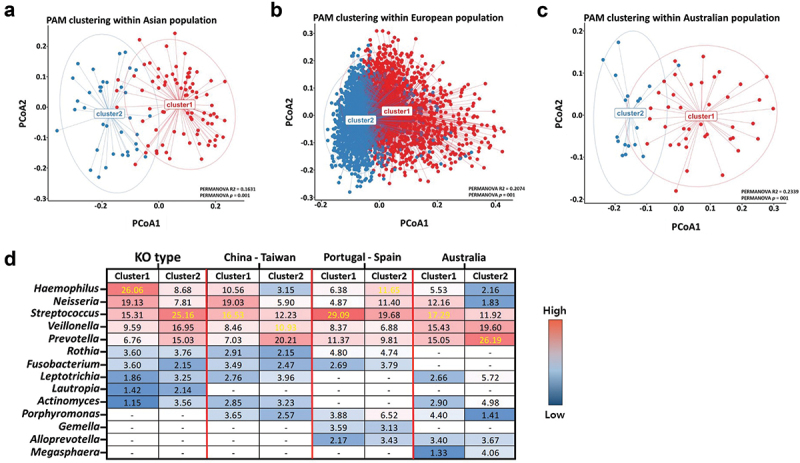
Comparison with oral microbiome data of other nations annotated on the public database.

**Note**: (a-c) The result of PAM clustering in Asian population (including Taiwan and China groups; *n* = 124), European population (including Portugal and Spain; *n* = 1341), and Australian population (*n* = 64) groups, respectively. (d) Heatmap plot showing the comparison of bacterial composition within salivary microbiome (only healthy control group) between each microbial cluster group (KO type vs. other countries corresponding to different living areas). The taxon names of the top 10 bacterial genera classified within each comparison group are shown on the vertical axis of the plot.

We also compared the clustered KO type to public data in other nations (China-Taiwan,_n = 124; Portugal-Spain,_n = 1341; and Australia,_n = 64) annotated in the National Center for Biotechnology Information (NCBI) database (Figure 3; Supplementary Table S5). To increase the reliability of this comparison, we applied raw data generated through 16S V3-V4 metagenomic sequencing of the saliva samples of healthy individuals without oral disease. Using the PAM clustering method, as with our microbiome type clustering results, we confirmed that the optimal microbial cluster numbers calculated within the composition data of the oral microbiome for each public data were two (Figure 3a,b,c). The major expected dominant bacterial genera that separated the optimal microbial clusters within each public dataset were as follows (Figure 3d): China-Taiwan (Asian), *Streptococcus* (Cluster 1) and *Veillonella* (Cluster 2); Portugal and Spain (European),_*Streptococcus* (Cluster 1) and *Haemophilus* (Cluster 2); and Australia (Oceanian),_*Streptococcus* (Cluster 1) and *Prevotella* (Cluster 2). Thus, *Streptococcus* was a common dominant bacterial genus that formed a microbial cluster within each public dataset. Although both the KO type and European (Portugal-Spain) data formed clusters around the *Streptococcus* and *Haemophilus* genera, we confirmed that there were differences in the bacterial composition (relative abundance) within each cluster group.

### Inference of bacterial compositional network within each oral microbiome type

We performed co-occurrence analysis to identify the compositional network (up to the second compositional network) between the bacterial taxa classified within each KO type (Table 2; Table 3; Supplementary Fig. S3). In this approach, the *Streptococcus* and *Haemophilus* genera, major dominant bacterial taxa for determining the characteristics of each KO type, became the criteria for verifying bacterial correlations. In the first network analysis, six (five positive and one negative) and five (three positive and two negative) significant correlations (|*R*| > 0.3 and *p* < 0.05) were found within the S and H types, respectively, from which 42 (33 positive and nine negative) and 30 (19 positive and 11 negative) significant (|*R*| > 0.3 and *p* < 0.05) correlations were identified through the second network analysis. In the first network of the S type, the *Granulicatella*, *Gemella*, and *Actinomyces* genera were strongly estimated to have a more positive correlation with *Streptococcus* than the others (*R* > 0.51). In contrast, the *Selenomonas* genus was deduced to have a negative correlation with *Streptococcus* (*R* = − 0.323). Interestingly, in the second network, the *Rothia* genus was deduced to have a strong positive correlation with all the genera positively correlated with *Streptococcus* in the first network except for the *Corynebacterium* genus. We also found that the *Streptococcus*, *Selenomonas*, *Gemella*, and *Granulicatella* genera are forming the potential correlation to affect bacterial colonization between each other within the oral cavity. In the H type, the relatively high positive correlations of *Veillonella* and *Megasphaera* genera with *Haemophilus* were found in the first network (*R* = 0.451 and 0.404, respectively). In contrast, *Treponema* and *Bacteroides* genera were deduced to have a negative correlation with *Haemophilus* (*R* < −0.32). In the second network of the H type, we found that many bacterial genera were linked to the second branch with *Streptococcus* compared to other genera in the first network (seven positive and eight negative correlations). Especially, it was confirmed that the *Gemella* and *Rothia* genera had a strong positive correlation with *Streptococcus* compared to the others (*R* = 0.810 and 0.682, respectively). In this network analysis, there were 10 and 9 distinct bacterial genera with statistically significant relative frequency differences between the two KO types in the significant co-occurrence networks identified within the S and H types (Supplementary Table S6). Even though we confirmed the compositional network within each KOGA type by applying the same co-occurrence analysis pipeline, we could not identify a significant correlation distinguished from the KO type. This result was presumed to be because the bacterial composition within each KOGA type was not significantly different from that of the upper-cluster group, the KO type. Therefore, we applied LEfSe analysis to identify distinct bacteria showing significant relative frequency differences between each KOGA type (Table 4). To observe various bacterial taxa, in which statistically significant LDA scores were calculated (logarithmic LDA score >2.0; *p* < 0.05), we identified the taxonomy by extending the range of the ranks to the species level in the genus. As a results of LEfSe analysis, total of nine distinct bacterial taxa were identified from each KOGA type.

**Table 2.** Compositional network between bacterial genera within S type.

KO type	1st network	Correlation	SparCC *R*-value	2nd network	Correlation	SparCC *R*-value
S type	*Granulicatella*	Positive (+)	0.562	*Rothia*	Positive (+)	0.609
				*Gemella*		0.582
				*Haemophilus*		0.359
				*Neisseria*		0.350
				*Selenomonas*	Negative (-)	−0.395
	*Gemella*	Positive (+)	0.529	*Granulicatella*	Positive (+)	0.582
				*Rothia*		0.536
				*Neisseria*		0.372
				*Corynebacterium*		0.304
				*Selenomonas*	Negative (-)	−0.509
				*Megasphaera*		−0.387
	*Actinomyces*	Positive (+)	0.517	*Rothia*	Positive (+)	0.621
				*Leptotrichia*		0.399
				*Neisseria*		0.335
				*Lachnoanaerobaculum*		0.330
				*Stomatobaculum*		0.328
				*Lachnospira*	Negative (-)	−0.323
				*UCG-002*		−0.303
	*Neisseria*	Positive (+)	0.357	*Rothia*	Positive (+)	0.538
				*Lachnoanaerobaculum*		0.387
				*Haemophilus*		0.378
				*Gemella*		0.372
				*Granulicatella*		0.350
				*Porphyromonas*		0.338
				*Actinomyces*		0.335
				*Peptostreptococcus*		0.333
				*Leptotrichia*		0.323
	*Corynebacterium*	Positive (+)	0.303	*Lautropia*	Positive (+)	0.446
				*Rothia*		0.390
				*Cardiobacterium*		0.374
				*F0332*		0.318
				*Gemella*		0.304
				*Amnipila*	Negative (-)	−0.302
	*Selenomonas*	Negative (-)	−0.323	*Alloprevotella*	Positive (+)	0.388
				*Atopobium*		0.373
				*Megasphaera*		0.336
				*Prevotella*		0.331
				TM7x		0.306
				*Capnocytophaga*		0.305
				*Gemella*	Negative (-)	−0.509
				*Granulicatella*		−0.395
				*Rothia*		−0.309

KO type: Korean oral microbiome type.S type: *Streptococcus*-dominant oral microbiome type.R: Correlation coefficient value.

**Table 3.** Compositional network between bacterial genera within H type.

KO type	1st network	Correlation	SparCC R-value	2nd network	Correlation	SparCC R-value
H type	*Veillonella*	Positive (+)	0.451	*Fusobacterium*	Positive (+)	0.509
				*Leptotrichia*		0.466
				*Prevotella*		0.434
				*Oribacterium*		0.413
				*Lachnoanaerobaculum*		0.393
				*Megasphaera*		0.307
				*Lautropia*	Negative (-)	−0.369
	*Megasphaera*	Positive (+)	0.404	*Veillonella*	Positive (+)	0.307
	*Streptococcus*	Positive (+)	0.377	*Gemella*	Positive (+)	0.810
				*Rothia*		0.682
				*Granulicatella*		0.377
				*Lactococcus*		0.357
				*Clostridium*-sensu-stricto-1		0.343
				*Amnipila*		0.343
				*Lachnospiraceae_*uncultured		0.304
				*Dialister*	Negative (-)	−0.421
				*Selenomonas*		−0.419
				*Alloprevotella*		−0.415
				*Bacteroides*		−0.347
				*Aggregatibacter*		−0.347
				*Prevotella*		−0.334
				*Lachnoanaerobaculum*		−0.310
				*Peptostreptococcus*		−0.301
	*Treponema*	Negative (-)	−0.322	*Lentimicrobium*	Positive (+)	0.370
	*Bacteroides*	Negative (-)	−0.321	*[Ruminococcus]-gnavus-*group	Positive (+)	0.441
				*Megamonas*		0.415
				*Blautia*		0.355
				*Escherichia-Shigella*		0.329
				*Streptococcus*	Negative (-)	−0.347
				TM7x		−0.315

KO type: Korean oral microbiome type.H type: *Haemophilus*-dominant oral microbiome type.R: Correlation coefficient value.

**Table 4.** Distinct bacterial taxa exported from LEfSe analysis between each KOGA type.

Bacterial taxa	Taxonomy rank	KOGA type	LDA score	Comparison groups	Kruskal–Wallis *p*-value	Statistical significance
*Enterococcus*	Genus	SP	2.72	SB vs. SP	0.002	**
*Granulicatella*	Genus	SB	3.37		0.017	*
*Capnocytophaga sputigena*	Species	SB	2.71		0.044	*
*Prevotella histicola*	Species	SP	3.63		0.034	*
*Prevotella veroralis*	Species	SP	2.83		0.033	*
*Prevotella*	Genus	HB	4.27	HB vs. HP	0.028	*
*Neisseria*	Genus	HP	4.65		0.008	**
*Prevotella melaninogenica*	Species	HB	4.16		0.008	**
*Veillonella atypica*	Species	HB	2.94		0.046	*

Statistical significance: *p < 0.05; **p < 0.01.

### Characterization of KOGA type

**Figure 4.**
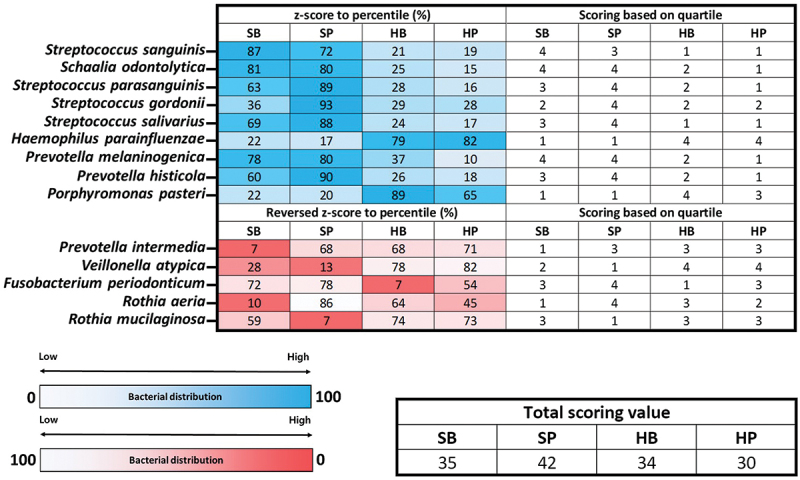
Heatmap plot and table showing the distribution of beneficial and harmful bacteria within oral samples of each KOGA type through scoring method.

**Note**: We visualized the distribution of selected beneficial or harmful bacterial species within the oral samples of each KOGA type through the heatmap plot applying *z*-score-based percentile (%) value and quartile score calculation results. The scoring criteria applied to convert the percentile value to the quartile score are as follows: 0 < % < 25: score 1, 25 ≤ % < 50: score 2, 50 ≤ % < 75: score 3, and 75 ≤ % < 100: score 4. The table indicates the total summation value of the quartile scores for each bacterial species within the KOGA type.

**Figure 5.**
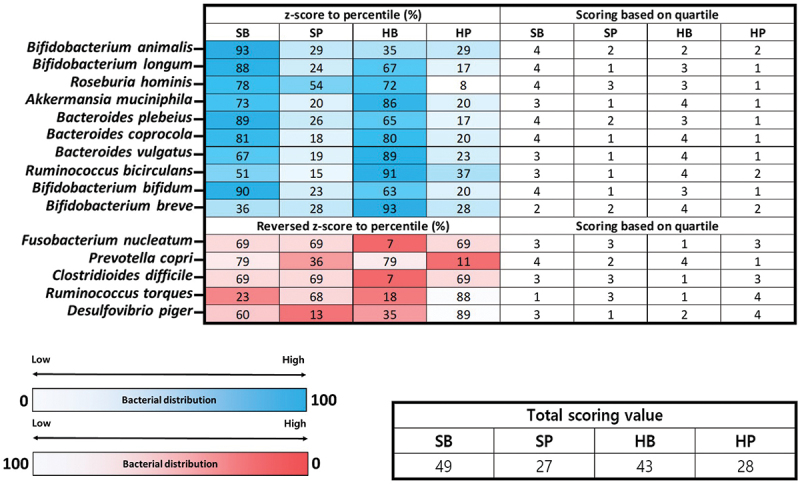
Heatmap plot and table showing the distribution of beneficial and harmful bacteria within gut samples of each KOGA type through scoring method.

**Note**: We visualized the distribution of selected beneficial or harmful bacterial species within the gut samples of each KOGA type through the heatmap plot applying *z*-score-based percentile (%) value and quartile score calculation results. The scoring criteria applied to convert the percentile value to the quartile score are as follows: 0 < % < 25: score 1, 25 ≤ % < 50: score 2, 50 ≤ % < 75: score 3, and 75 ≤ % < 100: score 4. The table indicates the total summation value of the quartile scores for each bacterial species within the KOGA type.

Finally, we estimated the potential health characteristics that could be described with microorganisms by confirming the distribution of beneficial, normal flora, and harmful bacterial species within the oral and gut samples of each KOGA type (Figure 4; Figure 5; Table 5). Unlike the previous process, we performed the analysis at the species level, considering that various bacterial species with different biological characteristics belong to the same genus level. We selected bacterial species (a total of 14 for oral and 15 for gut) that are well known to affect oral and intestinal health conditions, and the relative abundance data of each KOGA type was applied to confirm their distribution (Supplementary Table S7; Supplementary Table S8). The results of the percentile scoring converted from *z*-score for the normal distribution of relative bacterial frequency within each group were as follows (Detailed methods mentioned in ‘Materials and Methods’ section): In the analysis of the oral samples, the calculated potential health score within the SP-type was higher than that of the other types (the total scoring value of each KOGA type was SB 35, SP 42, HB 34, and HP 30). In the gut samples, a high potential health score was calculated for the two dominant *Bacteroides* types (the total scoring value of each KOGA type was SB 49, SP 27, HB 43, and HP 28). Based on the summation value of the scores within each KOGA type (oral and gut samples), the type with the highest calculated health score value was SB (summation scoring value of each KOGA type as was SB 84, SP 69, HB 77, and HP 58).

**Table 5.** Distribution of each bacterial species based on *z*-score calculation.

Information of selected bacterial species			SB	SP	HB	HP
Body sites	Bacterial characteristics	Bacterial species	*z*-Score			
Oral	Beneficial or normal flora	*Streptococcus sanguinis*	1.109359234	0.581684099	−0.821522451	−0.869520881
		*Schaalia odontolytica*	0.866037466	0.849673343	−0.690221094	−1.025489716
		*Streptococcus parasanguinis*	0.337667371	1.244063456	−0.581878978	−0.999851848
		*Streptococcus gordonii*	−0.358857274	1.492744777	−0.560460981	−0.573426521
		*Streptococcus salivarius*	0.491720027	1.165130406	−0.709880128	−0.946970304
		*Haemophilus parainfluenzae*	−0.776220496	−0.94881883	0.794166776	0.930872549
		*Prevotella melaninogenica*	0.770450127	0.826677921	−0.325488334	−1.271639714
		*Prevotella histicola*	0.245255701	1.30447429	−0.638181156	−0.911548835
		*Porphyromonas pasteri*	−0.78069542	−0.842878657	1.237271374	0.386302702
			Reversed_*z*-score			
	Harmful	*Prevotella intermedia*	−1.498508	0.4590254	0.477864	0.5616186
		*Veillonella atypica*	−0.5790756	−1.109888	0.7873802	0.9015834
		*Fusobacterium periodonticum*	0.5750737	0.7623781	−1.4412805	0.1038287
		*Rothia aeria*	−1.3043268	1.0787586	0.3468471	−0.121279
		*Rothia mucilaginosa*	0.2192554	−1.4718224	0.6508191	0.6017479
Body sites	Bacterial characteristics	Bacterial species	*z*-Score			
Gut	Beneficial or normal flora	*Bifidobacterium animalis*	1.494121361	−0.560581746	−0.372957869	−0.560581746
		*Bifidobacterium longum*	1.197021612	−0.703599839	0.442619949	−0.936041723
		*Roseburia hominis*	0.76553502	0.087938774	0.583712049	−1.437185843
		*Akkermansia muciniphila*	0.6107924	−0.848966561	1.088062043	−0.849887882
		*Bacteroides plebeius*	1.227955621	−0.638367168	0.380040967	−0.969629421
		*Bacteroides coprocola*	0.887645241	−0.90570478	0.843182545	−0.825123005
		*Bacteroides vulgatus*	0.427038072	−0.883200836	1.211329198	−0.755166434
		*Ruminococcus bicirculans*	0.033982266	−1.045271919	1.340779079	−0.329489427
		*Bifidobacterium bifidum*	1.262130174	−0.747183405	0.340924182	−0.855870951
		*Bifidobacterium breve*	−0.351455176	−0.570271686	1.491998548	−0.570271686
			Reversed_*z*-score			
	Harmful	*Fusobacterium nucleatum*	0.5	0.5	−1.5	0.5
		*Prevotella copri*	0.79944717	−0.36895163	0.8172482	−1.2477437
		*Clostridioides difficile*	0.5	0.5	−1.5	0.5
		*Ruminococcus torques*	−0.72759706	0.46694355	−0.9229054	1.183559
		*Desulfovibrio piger*	0.25744044	−1.11941193	−0.3780705	1.240042

## Discussion

Based on large-scale microbiome consortiums, such as HMP and MetaHIT, various human microbiome research has continuously been conducted to promote human health and prevent various systemic diseases [25,26]. In particular, many studies reported that it affects changes in the microbial composition inhabiting the inner or surface of various human body sites, depending on the individual’s living environment, lifestyle, clinical factors, medication use, and eating habits [27–29]. As the results of these studies were derived, recently, microbiome studies for determining the dominant microbial type representing each population were conducted to identify the differences in the human microbiome of each living area [22,30–32]. For example, approaches have been taken to cluster intestinal microbiome types (enterotype) highly affected by individual eating habits and then interpret the health status according to each type [33,34]. Although many studies have been conducted on type-clustering recently, research on the clinical variables (e.g. exposed various environments, individual hygiene habits, and surgery) affecting the oral microbiome composition is insufficient [35,36].

In our study, we identified the oral microbiome types of healthy Korean adults without oral diseases in association with the gut microbiome types and confirmed the potential health characteristics of each type through comprehensive microbiome analysis. Considering the reading of several hypervariable regions (V3-V4) on the 16S rRNA gene in this microbiome study, we minimized the error rate of the bacterial classification by identifying the microbiome type at the genus level. We confirmed that the two bacterial cluster types in the 112 healthy individuals were divided into *Streptococcus* (S-type) and *Haemophilus* (H-type) genera-predominant groups (KO type). *Streptococcus* and *Haemophilus* are well known as normal flora (or core microbiome), with high proportions in the human oral cavity [37–39]. Additionally, the four gut-associated oral microbiome types (KOGA type) were reclustered based on the individual’s gut enterotype (*Bacteroides* and *Prevotella* dominant types) corresponding to each KO type. We confirmed the difference between our clustering results and other oral microbiome studies conducted in various locations in different countries. Figure 3(a,b,c) show that the optimal microbial clustering number identified in all national oral microbiome data was two, as in our results. This result was the same as that of the HMP consortium analysis, which identified the salivary microbial composition of various human body sites [6,30]. From this comparison, we confirmed that the clustered oral microbiome type in the European samples was *Streptococcus* and *Haemophilus*-dominant. Still, there was a clear difference in the relative bacterial composition within each cluster type classified in the present study. We also suggest that although the major dominant bacteria in each cluster was different, our relative abundance data were somewhat similar to the oral microbiome composition classified from Asian samples. Based on these results, we could predict that our oral microbiome type clustering results showed some Korean specificity compared to other national data.

In co-occurrence analysis to identify compositional networks between bacterial genera, we found that normal flora in the oral cavity such as *Veillonella*, *Megaspaera*, *Granulicatella*, *Gemella*, *Actinomyces*, and *Neisseria*, formed positive correlations in the first network with *Streptococcus* and *Haemophilus* within each KO type [40–42]. In contrast, opportunistic infectious oral pathogenic bacteria, such as *Selenomonas* and *Treponema*, had negative correlations [43–45]. These results supported that the oral microbiome type clustering analysis was conducted on healthy groups without microbial-related oral diseases. Additionally, within the S type, we focused on the *Rothia* genus forms second networks with high positive correlation values with many bacterial genera that showed first correlations with the *Streptococcus*. Although *Rothia* is normal flora within the human oral cavity, it is opportunistic infectious bacteria with the potential to cause immune diseases in the host by producing enterobactin (a siderophore compound) [46,47]. Through the co-occurrence analysis, we could estimate the possibility of diseases caused by microorganisms within the S type. Additionally, we examined the bacterial compositional network in four KOGA types but found results almost similar to those of the upper cluster, the KO type.

Finally, we estimated the potential health characteristics for each KOGA type by confirming the *z*-score-based bacterial distribution of various beneficial and harmful bacterial species within the gut and oral samples. In this analysis, the SB-type was estimated to show the most potentially positive health effects in the oral and visceral samples of each KOGA type. Through this approach, we could simultaneously predict the potential health characteristics of the oral and gut for each KOGA type clustered in this study.

In summary, we clustered the oral microbiome types related to the gut enterotype through comprehensive 16S microbiome analysis of saliva and fecal samples collected from healthy Korean adults and identified the potential health characteristics. The present study was the first approach to identifying the oral microbiome types related to gut enterotypes and investigate their characteristics in healthy Koreans. Hence, we suggest that our results could be potential healthy control data for identifying differences in microbial compositions between healthy people and oral disease patients and studying microbial associations with the gut microbial environment (oral–gut microbiome axis).

## Supplementary Material

Supplemental Material

## Data Availability

The data that support the findings of this study are openly available in National Center for Biotechnology Information (NCBI) at [https://www.ncbi.nlm.nih.gov/bioproject/PRJNA940351/]. The annotation information of public data that support the findings of this study are openly available in National Center for Biotechnology Information (NCBI) at [https://www.ncbi.nlm.nih.gov/bioproject/?term=PRJEB39064, https://www.ncbi.nlm.nih.gov/bioproject/?term=PRJNA503603, https://www.ncbi.nlm.nih.gov/bioproject/?term=PRJNA609009, https://www.ncbi.nlm.nih.gov/bioproject/?term=PRJNA774299, https://www.ncbi.nlm.nih.gov/bioproject/?term=PRJNA427101, and https://www.ncbi.nlm.nih.gov/bioproject/?term=PRJNA558132].
