# Supplementary material for: Understanding the bacterial compositional network associations between oral and gut microbiome within healthy Koreans
Source: J Oral Microbiol. 2023 Mar 3;15(1):2186591. doi: 10.1080/20002297.2023.2186591 (PMC9987756; doi:10.1080/20002297.2023.2186591)
Supplement: Supplemental Material [file ZJOM_A_2186591_SM1954.zip › Supplementary files/Supplementary_information_final_ver..docx]

**Understanding the bacterial compositional network associations between oral and gut microbiome within healthy Koreans**

**Jinuk Jeong^1†^, Kung Ahn^2†^, Seyoung Mun^3,4^, Kyeongeui Yun^2,3^, Yeon-Tae Kim^5^, Won Jung^6^, Kyung Eun Lee^6^, Moon-Young Kim^7^, Yongju Ahn^2*^, and Kyudong Han^1,2,3,4,*^**

^1^ Department of Bioconvergence Engineering, Dankook University, Yongin 16890, Republic of Korea

^2^ HuNbiome Co., Ltd, R&D Center, Gasan digital 1-ro, Geumcheon-gu, Seoul, Korea

^3^ Department of Microbiology, College of Science & Technology, Dankook University, Cheonan 31116, Republic of Korea

^4^ Center for Bio‑Medical Engineering Core Facility, Dankook University, Cheonan 31116, Republic of Korea

^5^ Department of Periodontology, Daejeon Dental Hospital, Institute of Wonkwang Dental Research, Wonkwang University College of Dentistry, Daejeon, Korea

^6^ Department of Oral Medicine, School of Dentistry, Jeonbuk National University, Jeonju 54896, Korea, Department of Anesthesiology

^7^ Department of Oral and Maxillofacial Surgery, College of Dentistry, Dankook University,

Cheonan 31116, Korea

**Supplementary Data**

Figures S1-S3

Tables S1-S8

**Supplementary Figure legends**

**Supplementary Figure 1. Data processing for clustering KOGA type using PAM clustering method.**

This data processing shows the determination of four Korean oral-gut associated microbiome types (KOGA type) from each KO type using the PAM clustering method. (A) line graphs showing the results of the Partitioning Around Medoid (PAM) clustering method using the Calinéksi-Harabasz and Silhouette indexes based on the distance dissimilarity score of bacterial community between each individual within the two KO types calculated by applying the Jensen-Shannon divergence (JSD) formula. (B) Relative abundance bar plot showing the bacterial composition of each divided microbial cluster group. Bar graph and legend box on the plot show relative proportion and each taxon name of the top 10 bacterial genera in each cluster group.

**Supplementary Figure 2. Differential abundance plot of oral microbiome data by enterotype using MaAsLin 2**

Among the different genera in each clustering, *Haemophilus* (A) and *Streptococcus* (B) abundance were identified as high microorganism. In addition, the gut microbiome in each oral type were also identified (C, D, E and F) (*Bacteroides*, *Prevotella*). As an analysis option, relative abundance data created by QIIME2 was used as input data. (So, normalization and prevalence filtering about input data were set to 'None' parameter in MaAsLin 2 package).

**Supplementary Figure 3. Network plot showing bacterial correlation confirmed on bacterial composition within each KO type.**

(A) and (B) on the network plot are the results of confirming the bacterial compositional network linked to the major dominant bacterial genera (*Streptococcus* and *Haemophilus*) within the two different KO types (S and H types) through the Sparse Correlation Network Investigation for Compositional Data (SCNIC) analysis-based SparCC correlation methods. The blue and red lines on the plot mean positive and negative correlations between each bacterial genus. The sky blue and green oval shapes mean the first and second bacterial compositional networks identified in the correlation analysis.

**Supplementary Table lists**

**Supplementary Table 1. Clinical data and NGS statistics results of all participants in this study.**

**Supplementary Table 2. Bacterial taxonomy classification results with more 70% confidence threshold.**

**Supplementary Table 3. Relative abundance data processing about oral microbiome within control group for PAM clustering.**

**Supplementary Table 4. Relative abundance data processing about gut microbiome within control group for PAM clustering.**

**Supplementary Table 5. Comparison with oral microbiome data of other nations annotated on the public database.**

**Supplementary Table 6. Comparison of relative frequency of distinct bacterial genera in significant correlation.**

**Supplementary Table 7. Bacterial relative abundance of each KOGA type about oral and gut.**

**Supplementary Table 8. List of bacterial species selected for confirming distribution of beneficial and harmful bacteria.**
