# Supplementary figures and images for: Understanding the bacterial compositional network associations between oral and gut microbiome within healthy Koreans
Source: J Oral Microbiol. 2023 Mar 3;15(1):2186591. doi: 10.1080/20002297.2023.2186591 (PMC9987756; doi:10.1080/20002297.2023.2186591)

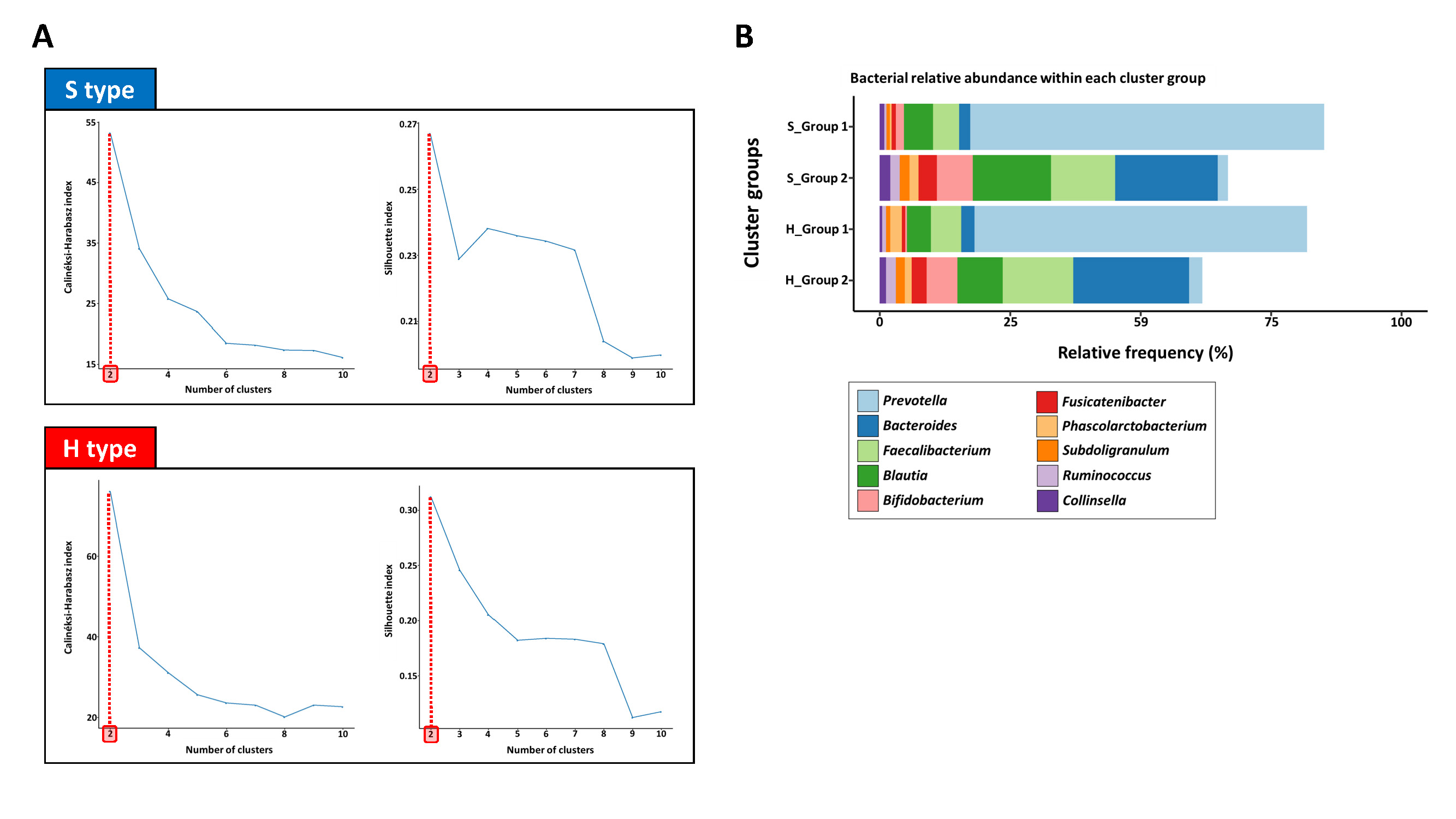

Supplement: Supplemental Material [file ZJOM_A_2186591_SM1954.zip › Supplementary files/Supplementary Figure 1.jpg]

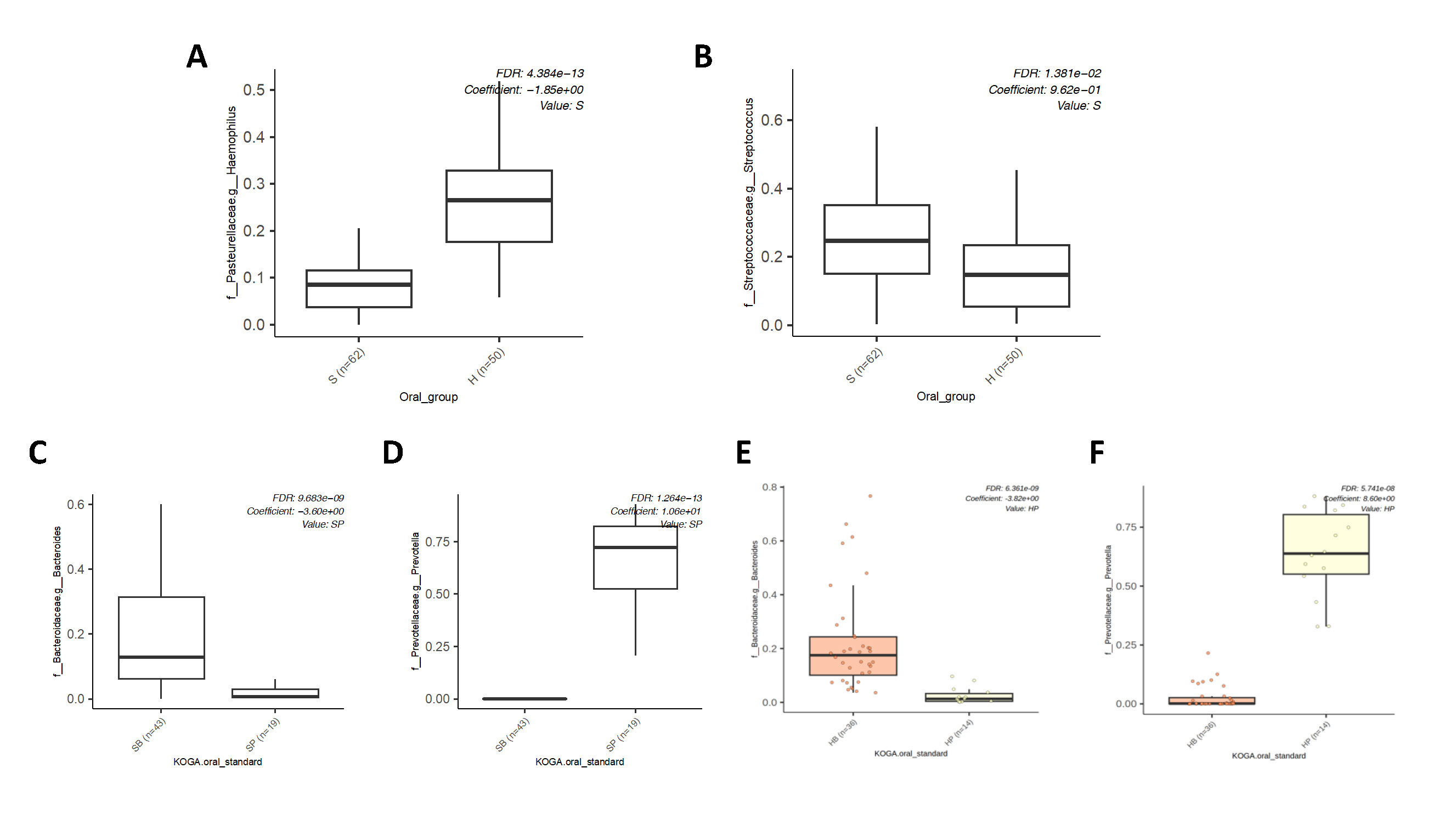

Supplement: Supplemental Material [file ZJOM_A_2186591_SM1954.zip › Supplementary files/Supplementary Figure 2.jpg]

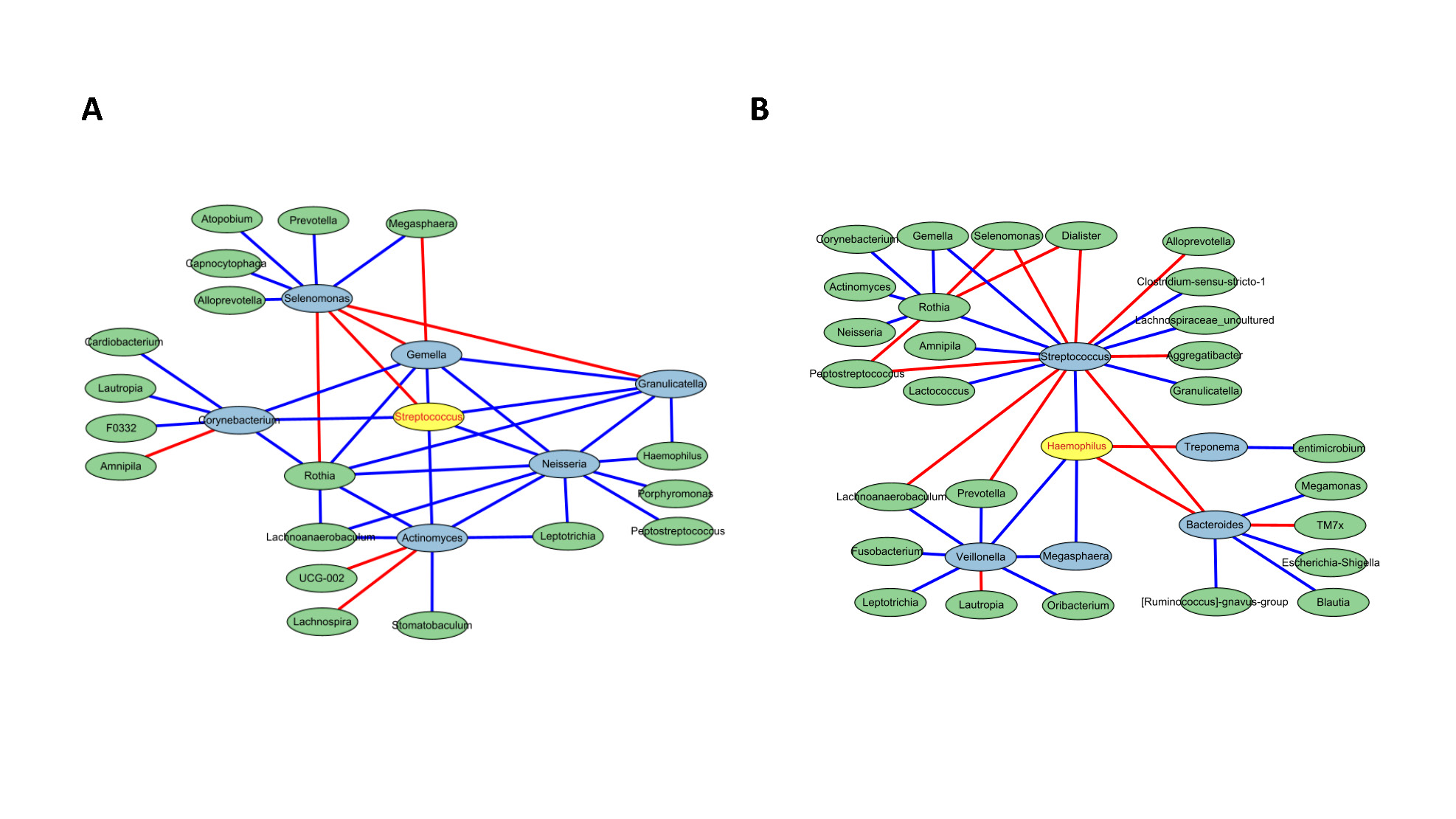

Supplement: Supplemental Material [file ZJOM_A_2186591_SM1954.zip › Supplementary files/Supplementary Figure 3.jpg]
